# Gender and Age Differences in Social Inequality on Adolescent Life Satisfaction: A Comparative Analysis of Health Behaviour Data from 41 Countries

**DOI:** 10.3390/ijerph15071297

**Published:** 2018-06-21

**Authors:** Apolinaras Zaborskis, Monika Grincaite

**Affiliations:** Medical Academy, Faculty of Public Health, Institute of Health Research and Department of Preventive Medicine, Lithuanian University of Health Sciences, LT-47181 Kaunas, Lithuania; monika.grincaite@lsmuni.lt

**Keywords:** adolescents, life satisfaction, social inequality, family affluence, HBSC

## Abstract

This study examined the gender and age differences in social inequality on life satisfaction among adolescents in 41 countries. Representative samples of adolescents aged 11–15 years from 41 countries were surveyed using the Health Behaviour Study among School-aged Children 2013/2014 (HBSC) questionnaire and research protocol. A Relative Index of Inequality (RII) estimated from the Poisson regression was employed to measure the level of social inequality. Family affluence was significantly positively associated with higher adolescent life satisfaction in nearly all countries (RII = 1.344; 95% confidence interval: 1.330–1.359). The average RII values for boys and girls were almost equal (1.318) and did not differ significantly across 11-, 13- and 15-year-old groups (1.373, 1.324 and 1.342, respectively). However, the gender and age differences in this association were controversial across countries. An analysis of data by countries revealed that among students from Israel, Italy and Ireland (in seven countries altogether), social inequality in life satisfaction (LS) was significantly greater for girls, while among students from Norway, the Republic of Moldova and the Czech Republic (in 20 countries altogether), social inequality in LS was significantly greater among boys; in 14 countries, the RII value did not significantly differ between boys and girls. Comparing social inequality in LS between 11- and 15-year-olds, in nine countries (e.g., Belgium-Flemish, Czech Republic, Netherlands) the RII was significantly greater among 15-year-olds, in 16 countries (e.g., Albania, the former Yugoslav Republic of Macedonia, Spain) the RII was significantly greater among 11-year-olds, and in 16 countries there were no significant differences in RII values. In conclusion, social inequality in adolescent LS differs between boys and girls and between age groups, providing substantial variation in these differences across countries.

## 1. Introduction

Social inequality, related to differences in, for example, income, employment and education, contributes to health inequality and most often put groups of low socioeconomic status (SES) at significantly higher risk for health problems, poorer quality of life and lower healthcare utilization [1,2]. Social inequality in health is one of the most commonly discussed topics worldwide and still remains one of the key health challenges of our time [3,4,5].

Although the impact of social determinants on adult health has dominated the research, examining this topic in early life has become more common [6]. The manifestation and consequences of social inequality in childhood and adolescence are the most unfair [7]. Children and adolescents are particularly vulnerable to the health-impairing effects of inequality as they are unlikely to possess or control much of the wealth or power held by society. There is evidence that family socioeconomic status (SES), including family affluence, is a primary determinant for young people’s social inequality and plays a crucial role in their health and well-being [8,9], this being of a major concern for public health and social policies in each country [10].

SES-based inequalities in health and health behaviours during adolescence are foci of the cross-national study on Health Behaviour in School-aged Children (HBSC) which involves a wide network of researchers from more than 40 countries and regions [11]. The previous editions of this study have highlighted the effects of socioeconomic differences on the way young people grow and develop [12,13]. A significant positive association between well-being and family affluence was found for both genders and for all three age groups (11-, 13- and 15-year-olds) in nearly all participating countries and regions. Adolescents from homes with high family affluence more frequently reported higher life satisfaction (LS), better health and less psychosomatic complaints than their peers from homes with lower family affluence. This apparent relationship has led to a more detailed study.

One of the first questions that arises when examining the problem of social inequality in health among young people is whether social inequality is manifested equally among boys and girls or among adolescents of different age groups. Thus, there is a need to define the groups of adolescents that are most vulnerable to social inequality.

Consistent findings from several studies have demonstrated that social inequality in health is more evident among girls than among boys; the girls stand out as having most ailments, for example, headaches or being far less satisfied with their appearance and self-image than boys are [14]. Meanwhile, the results of the HBSC study presented in its reports [8,9] allow us to assume that gender differences in social inequality for LS are heterogeneous across countries [12,13]. In a systematic review of socioeconomic inequalities and mental health problems in children and adolescents by Reiss (2013), no consistent gender patterns were derived from the reviewed studies [6].

Age is another factor that affects the magnitude of social inequality in health [15]. The transition from early to late adolescence may influence the relationship between family SES and adolescent well-being. During this transition period, a possible crucial time for changing behaviours and attitudes occurs at age 13 when the pubertal maturation process is the most advanced [16]. This process is a transformation from an asymmetrical relationship in early adolescence where adolescents are dependent on their parents into a more symmetrical one in late adolescence in which they are treated as more autonomous individuals [17]. This transition to greater independence and autonomy is likely to have more positive outcomes when family SES is a positive dimension of the process [18]. The literature presents a variety of data on age-related social inequalities in young people’s health. For instance, the ‘West of Scotland 11 to 16′ cohort study proposed that some specific dimensions of heath fluctuate during the transition period and would be influenced by young people from different social backgrounds mixing in schools and peer groups or within the youth culture, thus inequalities in these dimensions of health would be expected to diminish or disappear during this period [19]. Results from other studies did not support the above ‘equalization’ hypothesis and, on the contrary, found increasing inequalities across age groups of adolescents [20].

Increasing social and gender role pressure with growing age as well as restricted access to material resources and psychosocial strain have been already discussed as potential explanations for the observed health inequalities [15,21]. However, more research is needed to help gain insight into the variation in SES-based inequalities in well-being over the early life course. This, in turn, suggests that international comparisons have considerable analytic potential in this field of research.

The aim of this paper is to report family affluence-based inequality in adolescent life satisfaction among boys and girls and among adolescents of three age groups (11-, 13- and 15-year-olds) comparing these findings across 41 countries that conducted the HBSC survey in the 2013/2014 school year. Increasing the knowledge and understanding about how social inequality relates to adolescent well-being in different gender and age groups may contribute to the identification of the youth population groups that are the most sensitive to family affluence which is a key requisite for any health promotional attempts.

## 2. Materials and Methods

### 2.1. Subjects and Study Design

The data were obtained from the Health Behaviour in School-aged Children (HBSC) study, a cross-national survey with support from the World Health Organization (WHO, Europe) which was completed in 2013/2014 in 42 countries, including 40 European countries and regions (considered alone as countries, i.e., England, Scotland and Wales), Canada and Israel. More detailed background information about the study is provided in its website [11] and international report [13].

The population selected for sampling was 11-, 13- and 15-year old adolescents. Participants were selected using a clustered hierarchical sampling design, where the initial sampling unit was the school class. The data collection methods ensured that the samples of students were representative by age and gender. Specifically, the analyses presented here are based on the total number of 192,718 individual records (children with valid LS and Family Affluence Scale (FAS) data) obtained from 41 countries via the HBSC Data Centre (Bergen University, Norway). Table S1 (Supplementary Materials) indicates the samples by country (Armenia was excluded from the analysis due to deviance in the FAS assessment).

The data were collected by means of self-report standardized questionnaires. The surveys were administrated in school classrooms to ensure students’ confidentiality. Response rates at the school, class and student levels exceeded 80% in the majority of countries [13].

The study conformed to the principles outlined in the Declaration of Helsinki. National and local educational institutions agreed upon the study protocol. Ethical approval was obtained for each national survey according to the national guidelines and regulations at the time of data collection. Researchers strictly followed the standardized international research protocol to ensure consistency in survey instruments, data collection and processing procedures [22].

### 2.2. Measures

At the individual level, the outcome (dependent) variable was life satisfaction, and the explanatory (independent) variables were family affluence, family structure, gender and age.

*Life satisfaction (LS).* Students’ LS was rated using a measurement technique known as the Cantril (1965) ladder [23]. The revised technique has been shown to have a good reliability and convergent validity among adolescents aged between 11 and 15 years old [24]. The respondents were asked to take a look at the drawn ladder, with steps numbered from zero (“0”) at the bottom, to ten (“10”) at the top, with the instruction that the top of the ladder represents the best possible life, and the bottom of the ladder represents the worst possible life. Respondents were asked to indicate the step of the ladder at which they would place their life at present. Thus, the response was scored from 0 to 10 (an ordinary variable LS). Its inverse value (*Z* = 10 − LS) was used as an outcome variable in the model of health inequality measure in order to adopt the Poisson distribution. On average, the proportion of missing cases for LS was 3.7%.

*Family affluence* was measured by the Family Affluence Scale (FAS) which was specially developed for the international nature of the HBSC study [25,26]. The scale is simple and easy to answer, even for young adolescents. The FAS includes six questions with answers (assignment of points shown in parentheses): *Does your family own a car, van or truck?* “no” (0), “yes one” (1), “yes two or more” (2); *Do you have your bedroom for yourself?* “no” (0), “yes” (1); *During the past 12 months, how many times did you travel away on holiday (vacation) with your family?* “not at all” (0), “once” (1), “twice“ (2), “more than twice” (3); *How many computers does your family own?* “none” (0), “one” (1), “two” (2), “more than two” (3); *How many bathrooms (room with a bath/shower or both) are in your home?* “none” (0), “one” (1), “two” (2), “tree or more” (3); *Does your family have a dishwasher at home?* “no” (0), “yes” (1). A FAS score was calculated by summing the points to these six questions. Because the groups of respondents scored by 0–2 points and by 12–13 points were small they were combined into the FAS = 1 group and the FAS = 11 group, respectively. The groups of respondents that scored from 3 to 11 points were assigned to groups FAS = 2 through FAS = 10, correspondingly. On average, the proportion of missing cases in the total sample for FAS was 8.3%.

*Family structure*. To identify family structure, respondents were given a checklist to mark the people living in their home. Respondents were coded as living with “both parents” (0) if both the mother and father were ticked in a checklist or “not both parents” (1) in all other cases.

*Gender and age.* Equal proportions of adolescent boys and girls aged 11, 13 and 15 years were targeted for the study.

### 2.3. Statistical Analysis

All analyses were performed with SPSS (version 21.0; SPSS Inc., Chicago, IL, USA, 2012). They were conducted on data from all countries which included 192,718 individual records and on data within each country. Statistics were estimated with 95% confidence intervals (95% CI). Statistical tests with *p* < 0.05 were considered statistically significant. A sufficient condition for the significance of difference between two statistical estimations was considered to be the absence of overlapping confidence intervals.

An approach proposed by Kunst and Mackenbach (1995) was used to estimate the extent of SES-based inequality in health [27]. The Relative Index of Inequality (RII) which measures the impact of family affluence (SES) on adolescent LS was estimated from the Poisson regression. In this model, the ordinal FAS measure was transformed into a continuous *X* variable, scaled from 0 (the extreme lowest family affluence) to 1 (the extreme highest family affluence), incorporating appropriate population weights for each FAS category. The *X* variable was plotted on the horizontal axis, representing the cumulative distribution of the population for each FAS category. Then, the dependent variable (e.g., mean of LS score) was plotted at the midpoint of each FAS group proportion range. In the present study, the RII value denotes the ratio between the mean value of the inversed LS score (*Z* = 10 − LS) at the extreme highest FAS level (at *X* = 1) and at the extreme lowest FAS level (at *X* = 0). All models were adjusted for family structure variable and, where appropriate, for gender or age group variables. The goodness of fit of the model was controlled by the deviance value/df (its value from 0.5 to 2.0 was considered to be acceptable). The midpoints were calculated for each country separately. Analyses of the total sample of all countries were weighted by country sample size. The use of RII in adolescent LS analysis was described in our recent publication in detail [28].

## 3. Results

A total of 192,718 students from 41 countries were eligible for this study; 48.3% of them were boys and 51.7% were girls. A negligible range between gender proportions of was common in all countries, except for Ireland, for which the percentages of boys and girls were 37.7% and 62.3%, respectively. In the whole sample, the age groups of 11, 13 and 15 years achieved nearly equal proportions (31.0%, 35.0% and 34.0%, respectively). Across countries, there were nevertheless deviations, ranging from 23.8% to 39.9% in the youngest age group with similar patterns among 13-year-olds and 15-year-olds. In Slovakia, 11-year-olds were not surveyed.

Family structure distribution showed wide variation across countries, ranging from 54.5% of adolescents living with both parents in Greenland to 93.1% in Albania (73.5% in the total sample). The mean FAS ranged from 3.96 in Albania to 8.84 in Luxembourg; its average in the total sample was 6.92. The means of LS score ranged from 7.09 in Belgium (Flemish) to 8.26 in the Republic of Moldova; the average in the total sample was 7.61. The distribution of LS scores was found to be highly skewed and to not be normally distributed in all the countries and in the total sample (skewness = −1.028).

The impact of family affluence, family structure (living with both parents), gender and age on adolescent LS was assessed by a Poisson regression-based model (Table 1). The results of this analysis revealed a dominant effect of family affluence over family structure, gender and age, with these last variables also showing a significant effect on adolescent LS. The model had an acceptable goodness of fit (deviance value/df was equal 1.606). In addition, it was perceived that the estimation of the impact of family affluence on adolescents’ LS determines the RII. From the multivariate analysis, it follows that RII = 1.344. This indicates that adolescents from the least affluent families in comparison with those from the highly affluent families had a 34.4% higher inversed LS mean score. This is equivalent to a higher LS mean score being present among adolescents from more affluent families.

For every country, the proposed measure indicated that adolescents from more affluent families have higher life satisfaction. The results showed variation in the degree of inequality between countries (Supplementary Materials, Table S1). For example, the lowest inequality in LS was found among adolescents in Malta (RII = 1.088; 95% CI: 0.982–1.204), while the highest inequality in LS was found among adolescents in Hungary (RII = 1.777; 95% CI: 1.659–1.915). Relatively low estimations of social inequality were also revealed in samples of students from Belgium (Flemish and French) and high estimations were also revealed in samples of students from the Republic of Moldova and Israel.

Table 2 summarizes the above presented findings and also shows the average values of inequality in LS measured in groups of boys and girls as well as in three age groups and nominates three countries with the lowest and highest values of the RII in the corresponding groups.

**Figure 1:**
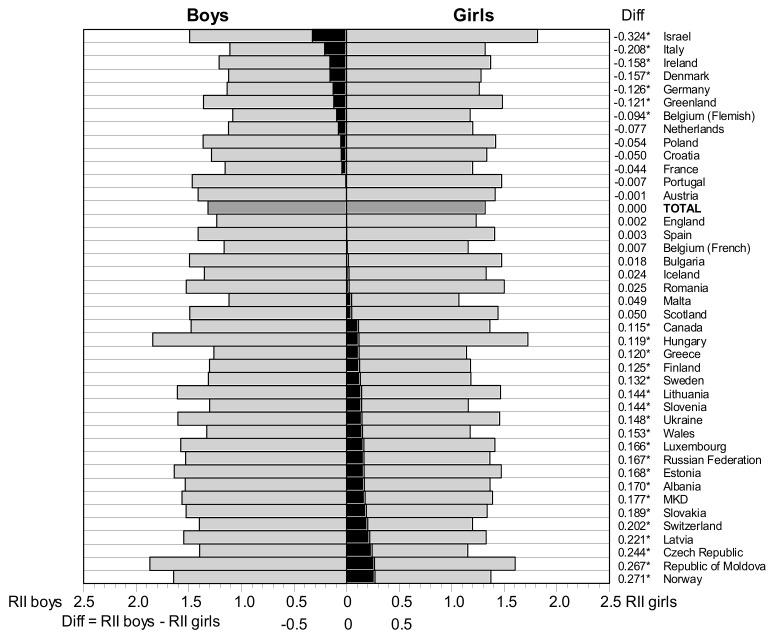
Relative Indices of Inequality (RII) in life satisfaction among boys and girls and their differences (black bars) in HBSC countries. * *p* < 0.05 that difference = 0.

In the total sample, almost equal RIIs (RII = 1.318) were found in groups of boys and girls which indicated no differences between gender groups for the influence of family affluence on adolescent LS. The analysis of data by country revealed significant gender differences in the effect of social inequality on LS among students in several countries; however, there was heterogeneity in differences (Figure 1). For instance, among students from Israel, Italy and Ireland (in seven countries altogether), the effect of social inequality on LS was significantly greater for girls, while among students from Norway, Republic of Moldova, Czech Republic (in 20 countries altogether), the effect of social inequality on LS was significantly greater among boys. In 14 countries, the RII value did not significantly differ between boys and girls.

**Figure 2:**
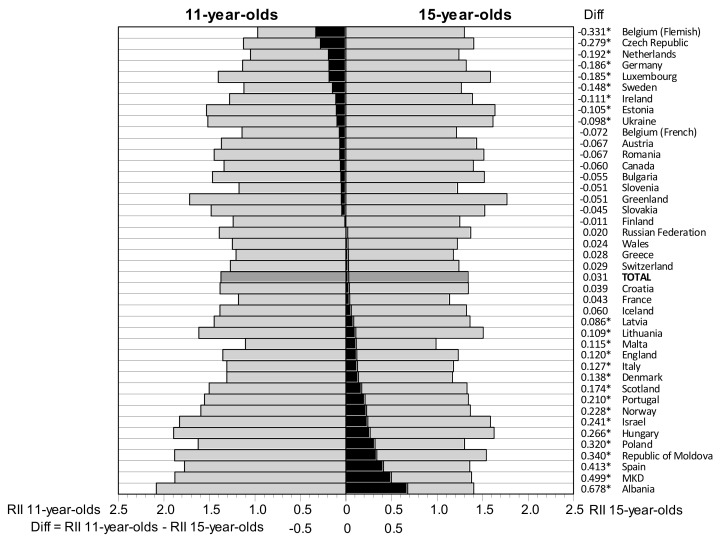
Relative Indices of Inequality (RII) in life satisfaction among 11-year-olds and 15-year-olds, and their differences (black bars) in HBSC countries. * *p* < 0.05 that difference = 0. For Slovakia, 11-year-olds were replaced by 13-year-olds.

With regard to the effects of age differences in social inequality on adolescent LS, analogous relationships were found. In the total sample, the RII values did not differ significantly between groups of 11-, 13- and 15-year-olds (1.373; 1.324 and 1.342 respectively). Across data from different countries, a heterogeneity in age group differences was observed. When social inequality in LS between 11- and 15-year-olds was compared, nine countries (e.g., Belgium-Flemish, Czech Republic, Netherlands) showed significantly greater RII values among 15-year-olds, in 16 countries (e.g., Albania, the former Yugoslav Republic of Macedonia (MKD), Spain), the RII was significantly greater among 11-year-olds, and in 16 countries, there were no significant differences in RII values (Figure 2).

## 4. Discussion

There is evidence that richer, wealthier or higher SES families provide better health and well-being among children and adolescents [8,9,14,29]. The present study aimed to answer whether inequalities in health vary between adolescent gender and age groups and, if so, whether the established regularities are common among all countries. A comparative analysis of health behaviour data from 41 countries revealed that social inequality in adolescent LS differs between boys and girls and between age groups; however, these differences vary substantially across countries.

In further analyses, we found that low family affluence as an indicator of low SES was associated with low adolescent LS. These results thus confirm the findings from other international studies, such as the KIDSCREEN survey [29]. A variety of mechanisms linking SES to child well-being and mental health have been proposed, with most focusing on the differences in access to material and social resources and, reactions to stress-inducing poverty by both the children themselves and their parents [21,30,31]. Still, the question remains as to whether these differences reflect true differences or how the health/well-being aspects are understood, conceptualised and expressed by respondents [15,21]. Validation studies have shown the FAS to function as a measure of SES and its results were largely comparable across the countries involved in the HBSC surveys [25,26]. Further studies are warranted as countries with lower SES status generally display higher prevalences of subjective health problems [31]. Our recent study revealed that higher income inequality (Gini Index) is associated with a higher inequality in adolescent LS [28]. Similar results were found in the KIDSCREEN study using the KIDSCREEN index of mental health and well-being [32]. All of the above provided studies revealed sizeable cross-national differences in the strength of association between SES and the subjective health indicators under study. However, the main pattern—older adolescents and girls (especially older girls) and low SES and their association with an increased risk for subjective health problems—was established for nearly all potentially possible countries. In this respect, our findings could provide the basis for further in depth examination of the roles of gender and age in the relationship between SES and adolescent LS.

Gender may be a contextual factor at the cultural level that explains cross-national differences as occurring due to different gender role traditions [33]. The findings of the HBSC studies [12,13] and other studies [14] demonstrated that girls stand out as having worse LS than boys. Gender differences seem to be geographically patterned [12,13]. We hypothesized, therefore, that social inequality due to differences in family affluence would tend to be greater among girls than among boys. However, the results of the present study on the HBSC showed that differences in social inequality go in slightly different directions for boys and girls in different countries. With regard to the LS, its social inequality due to differences in family affluence tended to be significantly greater among girls than among boys in seven countries, while a reverse association was observed among students of 20 countries. However, we did not find systematic patterns in the reasons for these gender differences based on geographical or socioeconomic, backgrounds.

Across data from the HBSC countries, a heterogeneity in social inequality of LS by age group was also observed. According to Chen et al. (2002) [34], three possible developmental models of the relationship between socioeconomic status (SES) and adolescent health can be taken in regard, when 11-year-olds are considered representatives of childhood and 15-year-olds are considered representatives of adolescence. The first model depicts the childhood-adolescent persistent model, showing that the SES and health relationship remains constant over time. In our study (see Figure 2), the data from 16 countries may apply to this model. The second model depicts the childhood-limited model, showing that the relationship between SES and health is strongest in childhood and weakens with age. The third model depicts the adolescent-emergent model, which shows that the relationship between SES and health is weak in childhood and becomes more pronounced with age. With regard to our study, the second and third developmental models of the relationship between family affluence and adolescent LS apply to data from 16 and 9 countries, respectively.

Age differences in the relationship between SES and young people health outcomes have been reported in several studies [15,19,20] but no consensus can be reached by comparing these studies. Although a variety of mechanism linking SES to child well-being and mental health have been discussed [21,31], the differences in results between studies could also be related to the indicators used to assess SES and health outcomes [15]. The national socio-economic situation, social support mechanisms, the educational system and the health service system are potential sources of variation in health inequalities between countries as well [21].

Compared with other studies in this field, the present study has several strengths. First, the study is the product of an international network of researchers who work in topic-focused groups that collaborate to research adolescent health. The international research protocol included scientific rational, international mandatory questions and required procedures for sampling, data collection and the preparation of data set to ensure high quality data. Second, in many studies, subjective health and well-being have been typically studied only in one or, at most, in a few countries. However, such investigations ignore an important cross-national source of variation. On the other hand, the use of a large, nationally representative sample and the inclusion of 41 countries increase the generalizability of our finding. Third, the analytical procedure based on RII and Poisson regression facilitates the comparative analysis of social inequality across countries, controlling for family structure and age (in assessing gender differences) or gender (in assessing age differences).

We hereby note several limitations of this study. First, this study relied only on self-reported data, although these data are considered to be the most valid when studying subjective feelings like LS [35]. The LS measure has been shown to have a good reliability and validity among adolescents aged between 11 and 15 years old [24]. Second, there are differences between countries in various aspects of data collection, and some of these might affect the size of inequalities in health and LS. The main concern was the family affluence assessment which was constructed by students’ self-reported items that are sensitive to the cultural and structural surroundings. Some studies have shown, however, that the FAS, that was used in our study, is a reliable measure among adolescents and is recommended in studies on health inequalities [25,26]. Third, unmeasured confounding may be an issue in this paper. The survey does not include measures such as parental education or occupation that are also associated with social inequality in adolescent well-being.

Despite these limitations, the present study provides an important contribution to public health research. Based on data from a collaborative cross-national survey and new analytical approach, the study highlighted the social inequality in adolescent LS and identified and discussed gender and age differences in social inequality across countries. The findings presented in this study can contribute to national health policies to reduce health inequalities by identifying the youth population groups that are most sensitive to family affluence in each country. These findings can also support the implementation of the European child and adolescent health strategy [36], which calls for targeted action to give every child an equal opportunity to live a healthy and happy life.

## 5. Conclusions

The association between family affluence and adolescent life satisfaction was found in all 41 HBSC countries, indicating that low family affluence is associated with low adolescent life satisfaction. The value of social inequality in life satisfaction differed between boys and girls as well as between age groups, providing substantial variation in differences across countries. These findings may contribute to the identification of the most sensitive youth population groups to family affluence in each country, which is considered to be crucial for adolescent health promotional attempts by national policy planners.

## Figures and Tables

**Table 1:** Gender, age, family structure and family affluence impacts on adolescent life satisfaction ^a^.

Variables and Categories	Univariate Analysis			Multivariate Analysis		
	Exp(B)	(95% CI)	*p*	Exp(B)	(95% CI)	*p*
Gender						
Boys	1			1		
Girls	1.133	(1.126–1.139)	<0.001	1.125	(1.118–1.132)	<0.001
Age group						
11-year-old	1			1		
13-year-old	1.283	(1.274–1.293)	<0.001	1.285	(1.275–1.295)	<0.001
15-year-old	1.465	(1.455–1.476)	<0.001	1.454	(1.443–1.465)	<0.001
Family structure						
Both parents	1			1		
Not both parents	1.270	(1.262–1.279)	<0.001	1.218	(1.210–1.226)	<0.001
Family affluence						
Extremely highest affluence	1			1		
Extremely lowest affluence ^b^	1.419	(1.405–1.434)	<0.001	1.344	(1.330–1.359)	<0.001

^a^ the model used inversed values (*Z* = 10–S). ^b^ estimations on this line represent the Relative Index of Inequality (RII). Exp(B): measure of variable impact on life satisfaction estimated in the Poisson regression model.

**Table 2:** Relative Index of Inequality (RII) in life satisfaction in the total sample and in selected countries, by adolescent group.

Group of Adolescents	RII Value (95% CI) in the Total Sample ^a^	RII Value (95% CI) in Selected Countries			
		3 Countries with the Lowest RII Value		3 Countries with the Highest Value	
All adolescents	1.344 (1.330–1.359)	Malta	1.088 (0.982–1.204)	Hungary	1.777 (1.659–1.915)
		Belgium (Flemish)	1.129 (1.058–1.205)	Republic of Moldova	1.736 (1.603–1.879)
		Belgium (French)	1.159 (1.092–1.231)	Israel	1.673 (1.554–1.801)
Boys ^b^	1.318 (1.303–1.333)	Belgium (Flemish)	1.082 (0.988–1.186)	Republic of Moldova	1.870 (1.671–2.094)
		Italy	1.110 (1.000–1.232)	Hungary	1.843 (1.653–2.055)
		Malta	1.118 (0.953–1.311)	Norway	1.644 (1.427–1.893)
Girls ^b^	1.318 (1.299–1.337)	Malta	1.069 (0.936–1.221)	Israel	1.816 (1.697–2.003)
		Greece	1.141 (1.034–1.259)	Hungary	1.724 (1.558–1.908)
		Czech Republic	1.153 (1.063–1.251)	Republic of Moldova	1.603 (1.433–1.792)
11-year-olds ^c^	1.373 (1.344–1.403)	Belgium (Flemish)	0.969 (0.863–1.089)	Albania	2.082 (1.784–2.430)
		Netherlands	1.049 (0.904–1.217)	Hungary	1.893 (1.650–2.172)
		Malta	1.104 (0.900–1.354)	Republic of Moldova	1.881 (1.612–2.193)
13-year-olds ^c^	1.324 (1.301–1.348)	Greenland	1.034 (0.779–1.373)	Hungary	1.845 (1.632–2.085)
		Belgium (Flemish)	1.050 (0.916–1.203)	Republic of Moldova	1.830 (1.600–2.094)
		Belgium (French)	1.116 (1.008–1.236)	Israel	1.628 (1.433–1.850)
15-year-olds ^c^	1.342 (1.320–1.366)	Malta	0.989 (0.835–1.171)	Greenland	1.768 (1.332–2.346)
		France	1.138 (1.029–1.259)	Estonia	1.637 (1.449–1.850)
		Denmark	1.170 (1.032–1.326)	Hungary	1.627 (1.434–1.846)

^a^ Data weighted by country/region sample size. Dependent variables in the regression model: ^b^ age group, family structure, family affluence; ^c^ gender, family structure, family affluence.

## Supplementary Materials

The following are available online at http://www.mdpi.com/1660-4601/15/7/1297/s1. Table S1: Effect of family affluence inequality on life satisfaction in gender and age groups of adolescents, by country.
