# Supplementary material for: Gender and Age Differences in Social Inequality on Adolescent Life Satisfaction: A Comparative Analysis of Health Behaviour Data from 41 Countries
Source: Int J Environ Res Public Health. 2018 Jun 21;15(7):1297. doi: 10.3390/ijerph15071297 (PMC6069104; doi:10.3390/ijerph15071297)
Supplement: Supplementary file 1 [file ijerph-15-01297-s001.pdf]

Table S1. Effect of family affluence inequality on life satisfaction in gender and age groups of adolescents, by countries.

Country	*n*	All adolescents [a]	Gender [b]		Age group [c]		
			Boys	Girls	11-year-olds	13-year-olds	15-year-olds
Albania	4718	1.450	1.534	1.364	2.082	1.137	1.404
Austria	3131	1.421	1.412	1.413	1.368	1.421	1.435
Belgium (Flemish)	5397	1.129	1.082	1.176	0.969	1.050	1.300
Belgium (French)	4006	1.159	1.164	1.157	1.142	1.116	1.214
Bulgaria	4379	1.484	1.493	1.475	1.465	1.443	1.520
Canada	11261	1.409	1.478	1.363	1.339	1.469	1.399
Croatia	6253	1.312	1.284	1.334	1.383	1.227	1.344
Czech Republic	4869	1.260	1.397	1.153	1.125	1.217	1.404
Denmark	5592	1.212	1.122	1.279	1.308	1.211	1.170
England	3550	1.230	1.234	1.232	1.353	1.139	1.233
Estonia	3959	1.549	1.639	1.471	1.532	1.452	1.637
Finland	4493	1.231	1.304	1.179	1.239	1.215	1.250
France	6627	1.177	1.155	1.199	1.181	1.216	1.138
Germany	5705	1.200	1.136	1.262	1.135	1.118	1.321
Greece	5243	1.199	1.261	1.141	1.207	1.213	1.179
Greenland	769	1.445	1.361	1.482	1.717	1.034	1.768
Hungary	4004	1.777	1.843	1.724	1.893	1.845	1.627
Iceland	4812	1.336	1.352	1.328	1.384	1.319	1.324
Ireland	3724	1.309	1.212	1.370	1.278	1.242	1.389
Israel	3623	1.673	1.492	1.816	1.828	1.628	1.587
Italy	4516	1.225	1.110	1.318	1.308	1.218	1.181
Latvia	9339	1.424	1.548	1.327	1.448	1.461	1.362
Lithuania	3908	1.531	1.609	1.465	1.616	1.478	1.507
Luxembourg	5487	1.484	1.577	1.411	1.402	1.392	1.587
Malta	2921	1.088	1.118	1.069	1.104	1.186	0.989
MKD	5401	1.468	1.565	1.388	1.879	1.301	1.380
Netherlands	4518	1.164	1.123	1.200	1.049	1.171	1.241
Norway	3916	1.476	1.644	1.373	1.594	1.508	1.366
Poland	2025	1.395	1.364	1.418	1.623	1.340	1.303
Portugal	3920	1.471	1.468	1.475	1.554	1.543	1.344
Republic of Moldova	2850	1.736	1.870	1.603	1.881	1.830	1.541
Romania	4190	1.516	1.525	1.500	1.448	1.534	1.515
Russian Federation	4368	1.431	1.530	1.363	1.391	1.519	1.371
Scotland	3518	1.474	1.491	1.441	1.503	1.603	1.329
Slovakia	4163	1.412	1.526	1.337	1.480	1.329	1.525
Slovenia	5611	1.219	1.302	1.158	1.174	1.250	1.225
Spain	7012	1.409	1.412	1.409	1.773	1.352	1.360
Sweden	4696	1.239	1.314	1.182	1.120	1.304	1.268
Switzerland	5218	1.283	1.400	1.198	1.270	1.337	1.241
Ukraine	4216	1.530	1.604	1.456	1.517	1.413	1.615
Wales	4810	1.245	1.329	1.176	1.247	1.269	1.223

Dependent variables in the regression model: [a] gender, age group, family structure, family affluence; [b] age group, family structure, family affluence; [c] gender, family structure, family affluence.
